# Molecular Prevalence and Genetic Diversity Based on *Msp1a* Gene of *Anaplasma ovis* in Goats from Türkiye

**DOI:** 10.3390/life13051101

**Published:** 2023-04-28

**Authors:** Mehmet Can Ulucesme, Sezayi Ozubek, Munir Aktas

**Affiliations:** Department of Parasitology, Faculty of Veterinary Medicine, University of Firat, Elazig 23119, Türkiye

**Keywords:** *Anaplasma ovis*, genetic diversity, goat, *MSP1a*, tandem repeats

## Abstract

*Anaplasma ovis* is a tick-borne obligated intraerythrocytic bacterium that infects domestic sheep, goats, and wild ruminants. Recently, several studies have been carried out using *16S rRNA* and *msp4* genes to identify the genetic diversity of *A. ovis*. Instead of these genes, which are known to be highly stable among heterologous strains, *Msp1a*, which is accepted as a stable molecular marker to classify *A. marginale* strains, was used in *A. ovis* genetic diversity studies. The genetic diversity of *A. ovis* strains according to the *Msp1a* gene has not been extensively reported. Therefore, the purpose of this study was to examine the genetic diversity of *A. ovis* in goats specifically using analysis of the *Msp1a* gene. Blood samples were taken from the vena jugularis to the EDTA tubes from 293 randomly selected goats (apparently healthy) in the Antalya and Mersin provinces of Mediterranean region of Türkiye. The *Msp1a* gene of *A. ovis* was amplified in all DNA samples through the use of PCR, using a specific set of primers named AoMsp1aF and AoMsp1aR. Among the amplified products, well-defined bands with different band sizes were subjected to sequence analysis. The obtained sequence data were converted into amino acid sequences using an online bioinformatics program and the tandem regions were examined. The *Msp1a* gene of *A. ovis* was amplified in 46.1% (135 out of 293) of the goats. Through tandem analysis, five distinct tandems (Ao8, Ao18, Tr15-16-17) were identified, and it was found that three of these tandems (Tr15-16-17) were previously unknown and were therefore defined as new tandems. The study also involved examination of ticks from goats. It was observed that the goats in the area were infested with several tick species, including *Rhipicephalus bursa* (888/1091, 81.4%), *R. turanicus* (96/1091, 8.8%), *Dermacentor raskemensis* (92/1091, 8.4%), *Hyalomma marginatum* (9/1091, 0.8%), and *R. sanguineus* s.l. (6/1091, 0.5%). This study provides important data for understanding the genetic diversity and evolution of *A. ovis* based on tandem repeats in the Msp1a protein.

## 1. Introduction

Microorganisms in the genus *Anaplasma* (family Anaplasmataceae, order Rickettsiales) are gram-negative obligate intracellular alphaproteobacteria. They are transtadially transmitted by ixodid tick species belonging to the *Rhipicephalus*, *Dermacentor*, *Ixodes*, *Amblyomma*, and *Haemaphysalis* genera to mammal hosts; they infect different blood cells (erythrocytes, monocytes, platelets, and granulocytes) in their hosts [1]. According to the taxonomic reclassification based on the *16S rRNA* and *groEL* genes, the genus *Anaplasma* comprised seven species: *Anaplasma marginale*, *A. centrale*, *A. phagocytophilum*, *A. bovis*, *A. ovis*, *A. capra*, and *A. platys* [1,2,3,4]. *Anaplasma ovis* is present in endemic countries including Türkiye and is known to infect sheep, goats, and wild ruminants [5,6,7,8,9] It is a major threat and worrying disease in almost all Mediterranean countries [10,11], Europe [12], Africa [13], and Asia [14,15]. A human case indicating the zoonotic potential of *A. ovis* has also been reported in Cyprus [16]. Although *A. ovis* is one of the rickettsial pathogens that cause common infection in farm animals, it has not been seriously evaluated [14]. The bacterium usually causes subclinical disease with a low-grade fever in sheep and goats [17,18]; however, under stress conditions, clinical disease with fever, hemolytic anemia, jaundice, anorexia, depression, lethargy, fatigue, and a decrease in milk production may occur [14,18,19,20,21]. A recent experimental study showed that *A. ovis* induces severe normocytic and normochromic anemia; however, evident clinical signs were not observed until the first lambing. Previous reports have indicated that lambs born from infected ewes did not show any clinical symptoms and only slight anemia with a rapid erythrocytic regenerative was observed [11]. Infection with *A. ovis* has been linked to an increased risk of contracting a variety of bacterial and parasitic diseases [22]. Ticks are the main transmission route for the bacterium, but cases of transmission through biting flies and exposure to fomites contaminated with infected blood have also been reported [12,23,24,25]. Animals that are able to survive acute anaplasmosis become chronically infected with the disease and thus act as reservoir hosts for the propagation of the agent [22,26].

There is a possibility that increases in virulence, pathogenicity, prevalence, and greater transmission are all associated with the increased genetic diversity of *Anaplasma* species that results in new strains [27,28,29]. Understanding the genetic diversity and different strains of *Anaplasma* species is essential to comprehending the epidemiology of the diseases they cause and improving their prevention and management. This is because anaplasmosis or tick-borne fever can be caused by various strains, and knowing the specific strain involved is critical in developing effective treatment strategies. In order to genetically identify *A. ovis* isolates, the *16S rRNA*, *gltA*, and *groEL*—in particular major surface proteins (MSPs) are analyzed [2,9,30,31,32,33]. There is evidence that the main surface proteins (MSPs) of the *Anaplasma* species play a role in interactions with both vertebrate and invertebrate hosts [34]. Since MSP1a’s N-terminal region contains a variable number of tandem amino acid repeats, it is more informative than other markers when it comes to detecting genetic diversity in *A. ovis* [7,15,33].

There is just one report of genetic diversity [7], despite the fact that sheep and goats in Türkiye have a high incidence of *A. ovis* (ranging from 18.3% to 89.3%) [35,36]. The purpose of this study was to investigate the prevalence of caprine anaplasmosis caused by *A. ovis* in the Mediterranean region of Türkiye and to determine the genetic diversity of *A. ovis* based on the *Msp1a* gene.

## 2. Materials and Methods

### 2.1. Study Locations and Design

**Figure 1:**
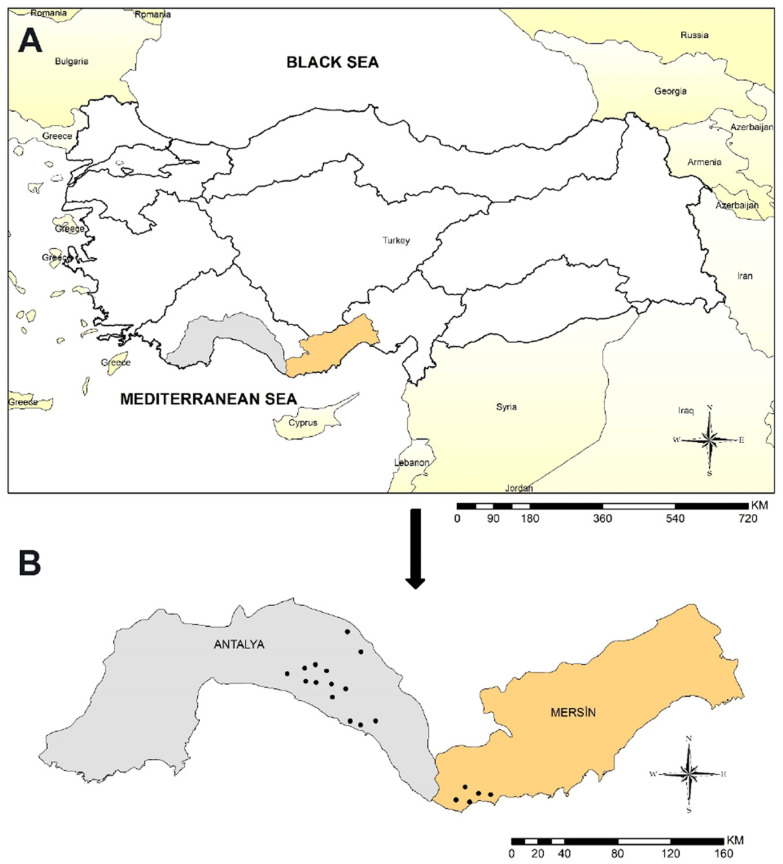
Map of Turkish provinces, indicating the localities analyzed in the study. (**A**) Geographical position of the provinces of Antalya and Mersin in Türkiye. (**B**) Position of localities sampled in the provinces of Antalya and Mersin.

A cross-sectional survey was conducted between May and September 2018 in the Antalya (latitude 36°53′ N, longitude 30°42′ E) and Mersin (latitude 36°47′ N, longitude 34°37′ E) provinces situated in the Mediterranean region of Türkiye (Figure 1). From both provinces, 24 private herds randomly selected from 19 different villages were visited to obtain the desired samples. The region has a climate typical of the Mediterranean, characterized by hot and humid summers, as well as warm and rainy winters. In Antalya, the average annual temperature and rainfall are 18.8 °C and 1061.7 mm, while in Mersin, they are 19.2 °C and 615.5 mm, respectively. In the region, traditional methods are used for breeding goats. During the winter months, the animals are kept in enclosed areas, while in the period from early spring to autumn, they are allowed to graze in pastures. The sample size was calculated using the online tool Sample Size Calculator (www.calculator.net/sample-sizecalculator.html, accessed on 10 March 2018), for a confidence level (CL) of 95% and an error margin of 5%. For the study, 7 to 36 goats were selected from each herd randomly based on the size of the herd, which ranged from 70 to 350 animals. The age of sampled goats was between 2 and 4 years. When the awareness of the farmers on the prevention of tick-borne infections was questioned, it was understood that regular acaricide treatment was not applied to the goats against the ixodid tick infestation.

### 2.2. Collection of Blood and Tick Samples

Sampling was performed from May to September 2018 in 19 villages situated in the Antalya and Mersin provinces. Two hundred and ninety-three apparently healthy goats were randomly sampled in one to two sampling herds from each village. Blood samples were collected into sterile 3 mL vacutainer EDTA (ethylenediaminetetraacetic acid) tubes from the jugular vein of the goats. The blood samples were kept in a cool box maintained at +4 °C and brought to the Department of Parasitology Laboratory, Firat University, Faculty of Veterinary Medicine, Türkiye and stored at −20 °C freezer until DNA extraction.

Sampled goats were also examined for the presence of ixodid tick infestations, and the status of tick infestations (presence or absence of ticks) were recorded. The ticks found on visible parts (ear, neck, abdomen, inguinal region) of the infested goats were removed using forceps and placed in 70% alcohol in 15 mL falcon tubes. The ticks were identified under stereomicroscope (SZX16, Olympus, Japan), according to the morphological characters described by [37,38]. During the study, a total of 1091 tick samples were collected from the goats. The sampled goats were grouped into categories according to the presence of ticks (yes/no). In this study, the ethics committee research protocol on animal use was approved by the Animal Ethics Committee of Ministry of Agriculture, Elazig Veterinary Control Institute (protocol number: 2018/02).

### 2.3. DNA Extraction, PCR Amplification and Reverse Line Blot (RLB) Hybridization

Blood samples were thawed at room temperature and vortexed for 10–15 s to homogenize, and DNA extraction was performed. Genomic DNA was isolated from 200 µL of the blood using the QIAamp DNA Blood Mini Kit (Qiagen, Hilden, Germany), according to the manufacturer’s instructions. As positive and negative controls, *Anaplasma ovis* DNA isolated from a naturally infected sheep (GenBank accession no. EU191232) and distilled water were used, respectively.

In order to amplify the *16S rRNA* gene of *A. ovis*, a nested PCR combined RLB was utilized, as was described in the previous literature [39,40]. In the first step of the process, outer (EC9/EC12A) and inner (16S8FE/B-GA1B-new) primers were utilized in nested PCR in order to amplify a 492–498-bp fragment of the *16S rRNA* gene. After that, 20 µL of nested amplicons obtained from each DNA sample were diluted with 2X SSPE 0.1% SDS buffer to a final volume of 150 µL. These diluted nested amplicons were then hybridized on an RLB membrane that had an *A. ovis* specific probe covalently linked to a Biodyne C blotting membrane (Pall Corporation). The primers and probes that were utilized for this study are presented in Supplementary Table S1.

### 2.4. Msp1a PCR

Samples that were positive for the *16S rRNA* gene of *A. ovis* were also screened by using a conventional PCR protocol with primer pairs AoMsp1aF and AoMsp1aR, which amplify the *Msp1a* gene [7,15]. Under ultraviolet light, amplicons were viewed to determine whether a band of the expected size (300–750 bp) was present after being deposited on an agarose gel containing 1.4% agarose that had been stained with SYBR Safe Gel Dye (Hibrigen, Türkiye). When determining whether different genotypes were present, the size of the bands produced on an agarose gel was one of the factors that was considered.

### 2.5. Sequencing, and Statistical Analysis

The amplified products of the *Msp1a* gene with a profile of strong, well-defined bands of varied sizes were chosen for sequencing. The QIAquick PCR Purification Kit was used to isolate twenty-one amplified fragments comprising varied *Msp1a* gene sequences (Qiagen, Hilden, Germany). The purified fragments were bidirectionally sequenced using the identical forward and reverse primers. Each construct was sequenced at least three times. The chromatograms were edited using Chromas Lite v. 2.1.1. (www.technelysium.com.au, accessed on 6 August 2022). The sequences were translated into proteins using Geneious v. 6.0.6, and the amino acid sequences were aligned using Clustal Omega Multiple Alignment (http://www.ebi.ac.uk/Tools/msa/clustalo, accessed on 6 August 2022). This study’s nucleotide sequences were deposited into the GenBank database and assigned accession numbers OL470941-OL470961. The statistical analysis of the data was performed using the SPSS 15.00 software program.

## 3. Results

### 3.1. Tick Infestation

Of the 293 goats examined for the presence of ixodid ticks, 194 (66.2%) were infested with at least 1 tick species. A total of 1091 adult ticks (330 females, 761 males) were collected from goats. The number of ticks per goat ranged from 1 to 49. The morphological identity of the ticks revealed that the sampled goats were infested with five different tick species. *Rhipicephalus bursa* (888/1091, 81.4%) was the dominant tick species, followed by *R. turanicus* (96/1091, 8.8%), *Dermacentor raskemensis* (92/1091, 8.4%), *Hyalomma marginatum* (9/1091, 0.8%), and *R. sanguineus* s.l. (6/1091, 0.5%). *Dermacentor raskemensis* were found to attach to the lower parts of the abdomen, while the other tick species were determined to attach to the ear and inguinal regions of the infested goats.

### 3.2. Anaplasma ovis Has Been Shown a High Prevalence in Field Samples

In total, 293 blood samples from goats were tested by nested PCR combined RLB for the presence of *16S rRNA* gene of *A. ovis*. Of the DNA-amplified products, 135 (46.1%, CI 40.3–52.0) showed a positive signal to the corresponding *A. ovis* specific probe. The infection was detected in all 19 villages, representing the 2 provinces included in this study. The prevalence of the bacterium varied from 6.7 to 93.8% in different sampling villages (Table 1). When the data of the two provinces were compared, it was evaluated as statistically insignificant (*p* > 0.05), but the relationship between the data of the villages was significant (*p* < 0.05).

Association of the prevalence of *A. ovis* in goats with tick infestation is presented in Table 2. The prevalence of *A. ovis* was comparable in tick infestation, and no difference was detected between infection rates in goats (*p* = 0.73133) (Table 2).

### 3.3. Three New Tandem Repeats Were Described in A. ovis MSP1a Protein Sequences

**Figure 2:**
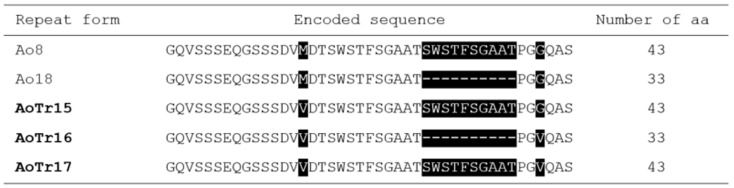
The MSP1a tandem repeat regions’ structure. Three novel Msp1a tandem repeat sequences, dubbed AoTr15-16-17, were discovered in *A. ovis* isolates from Türkiye. Variable amino acids are marked on a black backdrop, while spaces represent deletions/insertions.

A total of 135 samples signaling the *A. ovis*-specific probe were screened by PCR for the *MSP1a* gene, and 21 samples with strong, well-defined band profiles of varying sizes were chosen for sequence analysis. Amino acid sequences were obtained using Geneious from the DNA sequences obtained as a result of the sequence analysis (300–354 bp), and the tandem repeat structure in Msp1a amino acid sequences was analyzed by using multiple alignments. All *A. ovis* isolates obtained in this study had a one-amino acid repeat. A number of 5 distinct Msp1a repeats with 33 to 43 amino acids were determined. This study reported five tandem repeats named Ao8-GQVSSSEQGSSSDVMDTSWSTFSGAATSWSTFSGAATPGGQAS, Ao18-GQVSSSEQGSSSDVMDTSWSTFSGAATPGGQAS, AoTr15 (GQVSSSEQGSSSDVVDTSWSTFSGAATSWSTFSGAATPGGQAS), AoTr16 (GQVSSSEQGSSSDVVDTSWSTFSGAATPGVQAS), and AoTr17 (GQVSSSEQGSSSDVVDTSWSTFSGAATSWSTFSGAATPGVQAS). Out of these, three tandem repeats (AoTr15-16-17) were described for the first time in this study. The most common repeat sequence was Ao8, which was found in 10 of the 20 isolates. *Anaplasma ovis MSP1a* gene sequences (OL470941-OL470961) showed 99.7–100% sequence identity to the *Anaplasma ovis* isolate v3 (MG693756.1), *Anaplasma ovis* isolate hp4 (MG693738.1), and *Anaplasma ovis* isolate ada46 (MG693760.1) sequences isolated from goats in Türkiye (Figure 2, Table 3).

## 4. Discussion

Small ruminant anaplasmosis caused by *A. ovis* is prevalent across the world’s tropical and subtropical climates [7,14,15]. This study provided a thorough assessment of the prevalence, distribution, and genetic diversity of *A. ovis* in goats from Türkiye’s Mediterranean area. Here, a molecular survey was undertaken to determine the frequency of *A. ovis* in goats from the Mediterranean region of Türkiye. The finding revealed that the prevalence of *A. ovis* in the sampled animals was 46.1% (CI 40.3–52.0). The prevalence of *A. ovis* infection has previously been reported in small ruminants from central Anatolia [32] and southwestern Anatolia [14] with 31.4% and 60%, respectively, indicating that *A. ovis* is wide-spread in most regions of Türkiye. Compared to some Mediterranean regions, the prevalence in our study was lower than to the findings previously reported from Tunisia with 70.1% [41], Algeria with 78% [42], Corsica with 52% [12], and Sardinia with 81.8% [43]. Difference in reported prevalence rates may vary depending on many factors such as sampling procedure and size, a standardized assay, sampling season, type of farming, tick infestation status in the herd, and the degree of the exposure to tick vectors [41,42,44]. Our finding and the other published data indicate that infections caused by *A. ovis* are quite common all over the world. However, it is not clear what the impact of the disease is in terms of its clinical significance. It is supposed that *A. ovis* might be predisposed in the presence of other tick-borne infections [20,22]. Thus, in a recent study, *A. ovis* was found to be involved in approximately 80% of the mixed infections detected [45].

Tick-borne disease frequency and distribution are influenced by factors such as host, breed, age, sex, sample analysis methods, wildlife reservoir presence, farm management and husbandry practices, tick presence and abundance, and bioclimatic and ecological parameters [41,44,46]. We found no difference in prevalence rates between the two provinces studied because herd management, livestock practices, and bioclimatic and ecological parameters were all similar. The results of this study showed that an infestation of ticks did not increase the risk of infection in goats. This result did not come as a surprise due to the fact that the *Anaplasma* species is capable of being passed on by blood-feeding flies, contaminated syringes and other surgical equipment, as well as ticks [13]. In addition, the *A. ovis* was found to remain persistent in its host for more than twenty months following experimental infection [47]. This finding suggests that animals can harbor the *A. ovis* for a considerable amount of time, even in the absence of ticks.

The basis *gltA*, *16S rRNA*, and *msp4* genes have been the primary focus of research [19,46,48] on identifying the genetic diversity of *A. ovis*; however, it has been reported that these markers are conserved and are not sufficient to discover novel *A. ovis* genetic variants [2,30]. Msp1a has been utilized quite frequently as a molecular marker to characterize different strains of *A. marginale* due to the variable N-terminal region that contains repeating peptides [49]. It has been proposed that Msp1a developed as a response to the intensity of the immunological selection pressure, and that it varied between strains due to the varying sequences and quantities of tandem amino acid repeats that are present in the N-terminal region of the protein [30]. To this day, researchers from a variety of countries have identified a significant number of strains and genotypes containing hundreds of *A. marginale* Msp1a tandem repeats [30,49,50,51]. These strains and genotypes come from different nations. However, it has been emphasized that the *Msp1a* gene, which encodes the main surface protein 1a (Msp1a), should be utilized to better discriminate strains phylogeographically and to offer a more complete knowledge of the diversity and evolution of *A. ovis* isolates globally [7,15,33]. This would allow for a better understanding of the evolution of *A. ovis* isolates around the world. We found a relatively low number of Msp1a repeats of *A. ovis* in the region that we studied, and we identified five distinct Msp1a repeats there. Our findings were based on an analysis of the *Msp1a* sequence. As a result of this research, three of the five distinct Msp1a repeats were singled out for the very first time and given the designations Tr15-16-17. The Msp1a repeats that were discovered in this research were found to be highly variable, containing amino acids 33–43, and each isolate was found to contain exactly one repeat. Both of the remaining two tandem repeats, Ao8 and Ao18, were initially described in China in 2017 [15] and were also reported in Türkiye in 2018 [7]. It has been reported that the number of repeats of the *A. ovis Msp1a* gene ranges anywhere from one to four [7,15,33]. The genotypes of *A. ovis* were found in different foci, and it is speculated that these genotypes reflect the transfer and movement of ovine animals and vectors between these provinces.

A total of 58 tandem repeats have been reported so far, according to the *A. ovis Msp1a* sequences; of these, 24 have been found in China, 14 in Türkiye, and 20 in Tunisia. There will be confusion in naming tandem repeats as the number of *A. ovis Msp1a* sequences increases in different geographical regions [7,15,33]. (Supplementary Table S2). A piece of software known as Repeat-Analyzer has been developed in order to determine and catalog the tandem sequences of *A. marginale* that are responsible for serious infections in cattle [50]. Researchers investigating tandems for *A. marginale* can view all tandems in this program. If they find a new tandem, they can save it and give it a name in this section of the program. The *A. ovis Msp1a* gene was used in a genotyping study in Tunisia, and it was realized that different names were being used for the same tandem repeats (AoTn3-5 and AoTn4-12-14 have the same tandem sequences). In addition, the tandem repeat, which was given the name AoCg1 in the same study [33], was given the name AoTr2 in an earlier study carried out in Türkiye [7]. All of these misunderstandings can be avoided by developing a software program for *A. ovis* that is analogous to the RepeatAnalyzer software program that is utilized in *A. marginale*.

## 5. Conclusions

In this research, a high prevalence of *A. ovis* DNA was found in clinically healthy goats, and 5 distinct tandem repeats containing 33–43 amino acids were discovered. Three of these tandems are reported for the first time. Msp1a has the potential to be an informative marker for determining which *A. ovis* strains are present. There is a pressing need for additional research on the genetic diversity, evolution, and phenotypes of host–pathogen and vector–pathogen relationships, as well as the association of these strains with clinical infections.

## Figures and Tables

**Table 1:** *Anaplasma ovis* investigated in field-collected blood samples of goat from different provinces in Antalya and Mersin.

Location		No. Infected		
Province	Villages	No. Tested	No. Positive (%)	95% CI
Antalya	Elikesik	16	15 (93.8)	69.8–99.8
	Payallar	19	12 (63.2)	38.4–83.7
	Asmaca	20	13 (65.0)	40.8–84.6
	Gençler	20	8 (40.0)	19.1–63.9
	Güçlüköy	36	19 (52.8)	35.5–69.6
	Yarpuz	14	2 (14.3)	1.8–42.8
	Saraçlı	16	8 (50.0)	24.7–75.3
	Güzelyalı	14	10 (71.4)	41.9–91.6
	Yavrudoğan	7	2 (28.6)	3.7–71.0
	Gebece	9	2 (22,2)	2.8–60.0
	Susuzşahap	15	1 (6.7)	0.2–31.9
	Oymapınar	11	5 (45.5)	16.7–76.6
	Sevinçköy	10	1 (10.0)	0.3–44.5
	Yaylaalan	13	4 (30.8)	9.1–61.4
Mersin	Narince	20	5 (25.0)	8.7–49.1
	Derebaşı	10	4 (40.0)	12.2–73.8
	Çarıklar	20	16 (80.0)	56.3–94.3
	Emirşah	13	3 (23.1)	5.0–53.8
	Güneybahşiş	10	5 (50.0)	18.7–81.3
Total		293	135 (46.1)	40.3–52.0

**Table 2:** Association of the presence (*Msp1a* PCR-positive and negative) of *Anaplasma ovis* in goats with tick infestation.

		Presence of Ticks on the Goats	
		No	Yes
Number	293	99 (33.8%)	194 (66.2%)
Positive	135 (46.07%)	47 (34.8)	88 (65.2%)
Negative	158 (53.92%)	52 (32.9%)	106 (67.1%)
*p*-value		0.73133	

**Table 3:** MSP1a repeat form, number of repeat and amino acid (aa) variation, and BLAST analysis result in *A. ovis* isolates.

Isolates	Repeat Form	Number of Repeats	Number of aa	Accession Number	BLAST Analysis
Akseki1	Ao8	1	43	OL470956	99.7% *A. ovis* (MG693756)
Akseki2	Ao8	1	43	OL470959	99.4% *A. ovis* (MG693738)
Akseki53	Ao8	1	43	OL470947	100.0% *A.ovis* (MG693738)
Akseki65	Ao18	1	33	OL470946	98.6% *A. ovis* (MG693760)
Alanya13	Ao8	1	43	OL470949	100.0% *A. ovis* (MG693738)
Alanya14	Ao8	1	43	OL470961	100.0% *A. ovis* (MG693738)
Alanya39	Ao8	1	43	OL470941	99.4% *A. ovis* (MG693738)
Alanya49	AoTr15	1	43	OL470953	99.4% *A. ovis* (MG693738)
Alanya50	Ao8	1	43	OL470942	99.4% *A. ovis* (MG693738)
Alanya60	AoTr16	1	33	OL470944	97.5% *A. ovis* (MG693760)
Anamur7	Ao8	1	43	OL470957	100.0% *A. ovis* (MG693738)
Anamur10	AoTr16	1	33	OL470950	97.5% *A. ovis* (MG693760)
Anamur55	AoTr15	1	43	OL470951	98.7% *A. ovis* (MG693738)
Bozyazi26	AoTr15	1	43	OL470945	98.7% *A. ovis* (MG693738)
Bozyazi28	Ao18	1	33	OL470955	98.6% *A. ovis* (MG693760)
Cetin6	Ao8	1	43	OL470958	100.0% *A. ovis* (MG693738)
Manavgat83	AoTr15	1	43	OL470948	98.7% *A. ovis* (MG693738)
Manavgat89	Ao8	1	43	OL470960	99.4% *A. ovis* (MG693738)
Manavgat90	AoTr17	1	43	OL470952	98.7% *A. ovis* (MG693738)
Manavgat95	Ao8	1	43	OL470943	99.4% *A. ovis* (MG693738)
Manavgat130	Ao18	1	33	OL470954	98.6% *A. ovis* (MG693760)

## Data Availability

Data available in a publicly accessible repository.

## Supplementary Materials

The following supporting information can be downloaded at: https://www.mdpi.com/article/10.3390/life13051101/s1, Table S1: Nucleotide sequences of nested PCR primers and RLB probes used in the study. Table S2: The Msp1a amino acid repeat sequences of *A. ovis* different geographical strains identified from sheep and goat. Reference [52] are cited in Supplementary Materials.
