# Supplementary material for: Molecular Prevalence and Genetic Diversity Based on Msp1a Gene of Anaplasma ovis in Goats from Türkiye
Source: Life (Basel). 2023 Apr 28;13(5):1101. doi: 10.3390/life13051101 (PMC10223740; doi:10.3390/life13051101)
Supplement: Supplementary file 1 [file life-13-01101-s001.zip › life-2290787-supplementary.pdf]

Supplementary Table S1. Nucleotide sequences of nested PCR primers and RLB probes used in the study.

Primer	Sequence (5′-3′)	References
EC9	TACCTTGTTACGACTT	[52]
EC12A	TGATCCTGGCTCAGAACGAACG	[52]
16S8FE	GGAATTCAGAGTTGGATCMTGGYTCAG	[39]
B-GA1B	biotin-CGGGATCCCGAGTTTGCCGGGACTTCTTCT	[39]
AoMsp1aF	CGTTTCCATGTGCTACAATGCCG	[15]
AoMsp1aR	GCTGTTC GCTATCGCAGTCTGTG	[15]

Probs	Modification (5′-3′)	
Anaplasma/Ehrlichia catch-all	C6 amino-GGG GGA AAG ATT TAT CGC TA	[39]
A. ovis	C6 amino-ACC GTA CGC GCA GCT TG	[39]

Supplementary Table S2. The Msp1a amino acid repeat sequences of *A. ovis* different geographical strains identified from sheep and goat.

Repeat form	Encoded sequence	aa	Geographical location	References
Ao1	GQVSPSPSEHSSSSAVMDTSWSTFSGAATSWTSSGVATPGEQSSNQAS	46	China	[15]
Ao2	GQVSPSPSEHSSSSAVMDTSWSTFSGAATSWSTFSGAATPGEQSSNQAS	47		
Ao3	GQVSSSEHSSSSAVMDTSWSTFSGAATSWTSSGVATPGEQSSNQAS	46		
Ao4	GQVSSSEQGSSSAVMDTSWSTFSGAATSWSTFSGAATPGGQASNQAS	47		
Ao5	GQVSPSPSEHSSSSAVMDTSWSTFSGAATSWTSSGAATPGEQSSNQAS	46		
Ao6	GQVSSSAVMDTSWSTFSGAATSWSTFSGAATPGGQAS	37		
Ao7	GQVSSSEQGSSSAVMDTSWSTFSGAATSWTSSGVATPGGQAS	42		
Ao8	GQVSSSEQGSSSDVMDTSWSTFSGAATSWSTFSGAATPGGQAS	43		
Ao9	GQVSSSEHSSSSAVMDTSWSTFSGAATSWSTFSGAATPGGQAS	43		
Ao10	GQVSSSEQGSSSAVMDTSWSTFSGAATSWSTFSGAATPGGQAS	43		
Ao11	GQVSSSAVMDTSWSTFSGAATSWTSSGVATPGGQAS	36		
Ao12	GQVSSSEQGSSSDVMGTSWSTFSGAATSWSTFSGAATPGGQAS	43		
Ao13	GQVSSSEQGSSSYVMDTSWSTFSGAATSWSTFSGVATPGGQAS	43		
Ao14	GQVSSSEQGSSSDVMDTSWSTFSGAATSWSTFSGVATPGGQAS	43		
Ao15	GQVSSSEQGSSSYVMDTSWSTLSGAATPGGQAS	33		
Ao16	GQVSSSEQGSSPDVMDTSWSTFSGAATPGGQAS	33		
Ao17	GQVSPSPSEHSSSSAVMDTSWSTFSGAATSWTSSGVATPGGQAS	42		
Ao18	GQVSSSEQGSSSDVMDTSWSTFSGAATPGGQAS	33		
Ao19	GQVSSSEQGSSSAVMDTSWSTFSGAATPGGQAS	33		
Ao20	GQVSSSEQGSSSYVMDTSWSTFSGAATSWTSSGVATPGGQAS	42		
Ao21	GQVSSSDVMDTSWSTFSGAATSWSTFSGAATPGGQAS	37		
Ao22	GQVSSSEQGSSSDVMDTSWSTFSGAATPGEQSSNQAS	37		
Ao23	GQVSPSPSEHSSSSAVMDTSWTSSGAATPGEQSSNQAS	36		
Ao24	GQVSPSPSEHSSSSAVMDTSWSTFSGAATPGEQSSNQAS	37		
AoTr1	VQVSSSEQGSSSNVMDTSQSTFSGAATPGGQAS	33	Türkiye	[7]
AoTr2	GQVSSSEQGSSSYVMDTSWSTFSGAATPGGQAS	33		
AoTr3	GQVSSSGHSSSSAVMDTSWSTFSGAATPGEQSS	33		
AoTr4	GQVSSSEQGSSSDVMDTSWSTFSGAATSWSAFSGAATPGGQAS	43		
AoTr5	GQVSSSEQGSSSDVMDTSWSTFSGAATSWTFSGAATPGGQAS	42		
AoTr6	GQVSSSEQGSPSDVMDTSWSTFSGAATSWTSSGVATPGGQAS	42		
AoTr7	VQVSSSEQGSSSYVMDTSWSTFSGAATPGGQAS	33		
AoTr8	GQVSPSPSEHSSSSDVMDTSWSTFSGAATPGGQAS	33		
AoTr9	GQVSSSEQGSSSNVMDTSWSTFSGAATPGGQAS	33		
AoTr10	GQVSSSEQGSSSDVMDTSWSTFSGAATSWTSCGAATPGGQAS	42		
AoTr11	GQVSSSEQGSSSDVVDTSWSTFSGAATSWTFSGAATPGVQAS	42		
AoTr12	GQVSSSEQGSSSDVMDTSWSTLSGAATPGGQAS	33		
AoTr13	GQVSSSEQGSSSYVMDTSWSTFSGAATPGGFSGAATPGGQAS	42		
AoTr14	GQVSSSEQGSSSDVMDTSWSTFSGAATPGGFSGAATPGGQAS	42		
AoTn1	DQVSSSEQVSSSEQGSSSDVMDTSWSTFSGAATSWTFSGAATPGGQAS	48	Tunisia	[33]
AoTn2	DQVSSSGQVSSSEQGSSSDVMDTSWSTFSGAATSWTFSGAATPGGQAS	48		
AoTn3-5	DQVSSSGQVSSSEQGSSSDVMDTSWSTFSGAATSWSTFSGAATPGGQAS	49		
AoTn4-12-14	DQVSSSEQGSSSDVMDTSWSTFSGAATSWTFSGAATPGGQAS	42		
AoTn3-5	DQVSSSGQVSSSEQGSSSDVMDTSWSTFSGAATSWSTFSGAATPGGQAS	49		
AoTn6	DQVSSSGQVSPSPSEHSSSSAVMDTSWRTFSGAATSWSTFSGAATPGGQAS	49		
AoTn7	DQVSSSGQVSSSEHSSSSDVMDTSWSTFSGAATSWSTFSGAATPGGQAS	49		
AoTn8	DQVSSSEQGSSSDVMDTSWSTFSGAATSWSTFSGAATPGGQAS	43		
AoTn9	DQVSSSDQVSSSEQGSSSDVMDTSWSTFSGAATSWTFSGAATPGGQAS	48		
AoTn10	DQVSSSEQGSSSDVMDTSWSTFSGAATSWTSSGAATPGGQAS	42		
AoTn11	DQVSSSGQVSSSEQGSSSDVMDTSWSTFSGAATSWSTFSGAATPGGQTS	49		
AoTn4-12-14	DQVSSSEQGSSSDVMDTSWSTFSGAATSWTFSGAATPGGQAS	42		

AoTn13	DQVSSSEQVSSSEVGDSSGSTFGGAATSWTSSGAATPGGQAS	42
AoTn4-12-14	DQVSSSEQGSSSDVMDTSWSTFSGAATSWTFSGAATPGGQAS	42
AoTn15	DQVSSSEQVSSSEQGSSSDVMETSWSTSSGAATSWSTFSGAATPGGQAS	49
AoTn16	DQVISSQQGSSSHVMDTSRSTFTGAATSWTSSGAPTPRGQAS	42
AoTn17	GQVSSSEQGSSSDVVDTSQSTFSGAATPGGQAS	33
AoTn18	GQVSSSGQVSSSEQGSSSDVMDTSWTLSGAATPGGQAS	38
AoCg1/AoTr2	GQVSSSEQGSSSYVMDTSWSTFSGAATPGGQAS	33
AoCg2	GQVSSSGQVSSSEQGSSSYVMDTSWSTFSGAATPGGQAS	39
